# Regional anesthesia after spinal cord injury attenuates behavioral loss, hemorrhage, and cell death

**DOI:** 10.3389/fneur.2026.1880792

**Published:** 2026-09-01

**Authors:** Natsuka Kobayashi, Jacob A. Davis, Calvin T. Phinney, James W. Grau

**Affiliations:** 1 Behavioral and Cellular Neuroscience, Department of Psychological & Brain Sciences, College Station, TX, United States; 2 Weill Institute for Neurosciences, Department of Neurological Surgery, University of California, San Francisco, San Francisco, CA, United States

**Keywords:** anesthesia, bupivacaine, cell death, hemorrhage, spinal cord injury

## Abstract

**Objective:**

Prior research has shown that regional application of an anesthetic (lidocaine) via a lumbar puncture can attenuate pain-induced secondary tissue loss after spinal cord injury (SCI). Applying lidocaine rostral to injury (at T2) also had a protective effect, suggesting neural signaling to/from rostral systems contributes to pain-induced tissue loss after SCI. The present study examined whether regional anesthesia protects the injured spinal cord when applied immediately after injury in the absence of additional pain. We hypothesized that inhibiting cellular activity in the region of injury would have a protective effect and that increasing the duration of treatment (from 1.5 to 12 h) would amplify this outcome.

**Methods:**

Male rats received a severe T12 contusion injury and were fitted with a catheter with the tip positioned either at L4 (Experiment 1) or at T2 (Experiment 2). Infusion of a marker at L4 showed dispersion extending to the site of injury. Infusion at T2 was restricted to the upper thoracic region. Regional anesthesia was induced using bupivacaine due to its wide use and long duration of action. Immediately after injury, animals received 30 μL of 0.75% bupivacaine or saline through the implanted catheter. Separate groups then received 0, 1, 3, or 7 additional infusions at 90 min intervals, yielding four treatment conditions: 1.5, 3, 6, or 12 h of local anesthesia. A day after injury, locomotor performance was assessed and a 1 cm region of injured spinal cord was taken to be assayed for markers of hemorrhage, programmed cell death, and inflammation.

**Results:**

Animals that received bupivacaine rostral to injury for 3–12 h exhibited improved locomotor recovery, less hemorrhage, and reduced cell death at the site of injury. Treatment at L4 was less effective in attenuating hemorrhage and cell death. In both experiments, there was no indication that increasing the duration of treatment beyond 3 h had added benefit.

**Conclusion:**

The results suggest that local application of an anesthetic at, or rostral to, a SCI may have a protective effect.

## 1 Introduction

Tissue loss after spinal cord injury (SCI) stems from the initial mechanical insult to the tissue (primary injury) and the processes that unfold over hours to days afterward (secondary injury) (1). Prior work has shown that engaging pain (nociceptive) fibers a day after injury using intermittent noxious electrical stimulation applied caudal to injury (to the tail) or capsaicin applied to one hind paw increases secondary tissue loss and impairs long-term recovery (2, 3). This effect has been linked to the breakdown of the blood-spinal cord barrier (BSCB) and the infiltration of blood (hemorrhage) into the site of injury (4, 5). This hemorrhage leads to inflammation and engagement of signal pathways (e.g., caspase-3) that drive cell death (6).

Recognizing that SCI is often accompanied by additional tissue damage (polytrauma) that would engage nociceptive fibers (noxious stimulation), we sought to identify treatments that could counter pain-induced tissue loss after injury (3). Pretreatment with an opiate analgesic (morphine) did not reduce hemorrhage or counter the effects of noxious stimulation on long-term recovery (7, 8). Instead, morphine had an adverse effect that increased tissue loss and mortality (9). In contrast, regional application of an anesthetic (lidocaine) prior to noxious stimulation, administered by means of a lumbar (L5) puncture, blocked nociception-induced hemorrhage, tissue loss, and impairment in long-term recovery (10).

Our studies have used a moderate contusion injury, which preserved some ascending/descending fibers to/from the brain. To evaluate whether these preserved pathways play a role, we examined whether an upper thoracic (T2) spinal cord transection affects nociception-induced hemorrhage in contused rats (11). Surprisingly, the application of a noxious stimulation caudal to a T12 contusion injury did not induce hemorrhage, inflammation, or caspase-3 expression in animals that also received a T2 transection. Further work showed that a pharmacological transection, produced by slowly infusing lidocaine at T2, had an equivalent protective effect and attenuated deficits in long-term recovery produced by noxious stimulation (12). Sedating animals with pentobarbital prior to noxious stimulation also attenuated hemorrhage, inflammation, and cell death (13). These findings suggest that rostral (brain-dependent) processes contribute to secondary tissue loss after SCI.

Our experiments to date have explored how anesthetic treatments, applied prior to noxious stimulation 24 h after injury, affect secondary tissue loss and long-term recovery (10, 12, 13). In these experiments, application of the anesthetic did not have a significant effect on hemorrhage or locomotor recovery in the absence of pain. It is possible that differences were not seen without noxious stimulation because secondary injury processes linked to the initial insult have already had time (24 h) to unfold. Perhaps regional anesthesia would have a protective effect if initiated *immediately* after injury. Such an outcome would suggest a new treatment that could be rapidly transitioned to clinical practice to reduce cell death and hemorrhage at the site of injury. The current study explores this possibility using the voltage-gated sodium channel (Nav) blocker, bupivacaine. Like lidocaine, bupivacaine impedes the inward flow of sodium into nerve cells and thereby blocks neural conduction. Bupivacaine was selected because it is much more potent than lidocaine and has a longer duration of action (14). While new drugs (e.g., levobupivacaine, ropivacaine) have improved safety, bupivacaine is more widely used world-wide (15), enhancing translational significance.

Because prior work found lidocaine applied to the lumbosacral region spread to the site of injury and attenuated pain-induced tissue loss after SCI (10), we hypothesized that bupivacaine applied to this area soon after injury would reduce hemorrhage and inflammation, and improve locomotor recovery (Experiment 1). We further hypothesized that the magnitude of these effects would grow with increased treatment duration (from 1.5 to 12 h). A second experiment was performed to assess whether bupivacaine applied rostral to injury (at T2) has a protective effect (Experiment 2). In the absence of noxious stimulation, we assumed that the processes that drive hemorrhage and inflammation after injury depended solely upon local mechanisms, not rostral systems. For this reason, we predicted that bupivacaine administered rostral to the site of injury would have little effect. We show that applying bupivacaine for 3 or more hours has a protective effect and that contrary to our original hypothesis, the greatest benefit was achieved when the drug was applied rostral to injury.

## 2 Materials and methods

### 2.1 Animal subjects

Ninety-six adult male Sprague Dawley rats (100–120 days old, 300–500 g) were obtained from Envigo in Houston, TX for this study. Rats were pair-housed under a 12-h light–dark cycle with *ad libitum* access to food and water. Animals were housed for at least 7 days before being acclimated to behavioral assessment procedures. Experiments were performed during the light cycle. All experiments were approved by Texas A&M University Institutional Animal Care and Use Committee and were performed in accordance with the National Institute of Health (NIH) standards for the care and use of laboratory animals.

### 2.2 Surgery

To induce anesthesia, rats were given a 5% isoflurane and medical oxygen mixture at a flow rate of 1 L/min. Once a stable anesthetic state was achieved, animals were maintained on 3% isoflurane given through a nose cone during surgery. An initial 3 cm longitudinal incision was made on the midline of the back and surrounding tissue was removed to reveal the spinous process of the vertebrae. A laminectomy was then performed to expose the spinal cord at the T11-12 level. The MASCIS device (NYU Medical Center, New York City, NY) was used to fix the spinal cord and provide a severe contusion injury by dropping a 10 g weight from a height of 25.0 mm directly onto the exposed cord. Immediately after, an 18.5 cm long polyethylene cannula (PE-10), Scientific Commodities, Lake Havasu City, AZ) was inserted into the epidural space and threaded either 4.0 cm caudal or 4.5 cm rostral to the site of injury to target L4 and T2, respectively. The catheter was secured using super glue and sutures. Catheterization was chosen over a puncture technique to minimize repeated invasive drug injections. The surgical site was closed with Michel clips. Animals were administered 3 mL of saline intraperitoneally to compensate for any blood loss and 100,000 units/kg of penicillin to prevent infection. For 24 h post-surgery, all rats were allowed to recover with free access to food and water in a temperature-controlled room, and bladders were manually expressed every 8–12 h.

**Figure 1:**
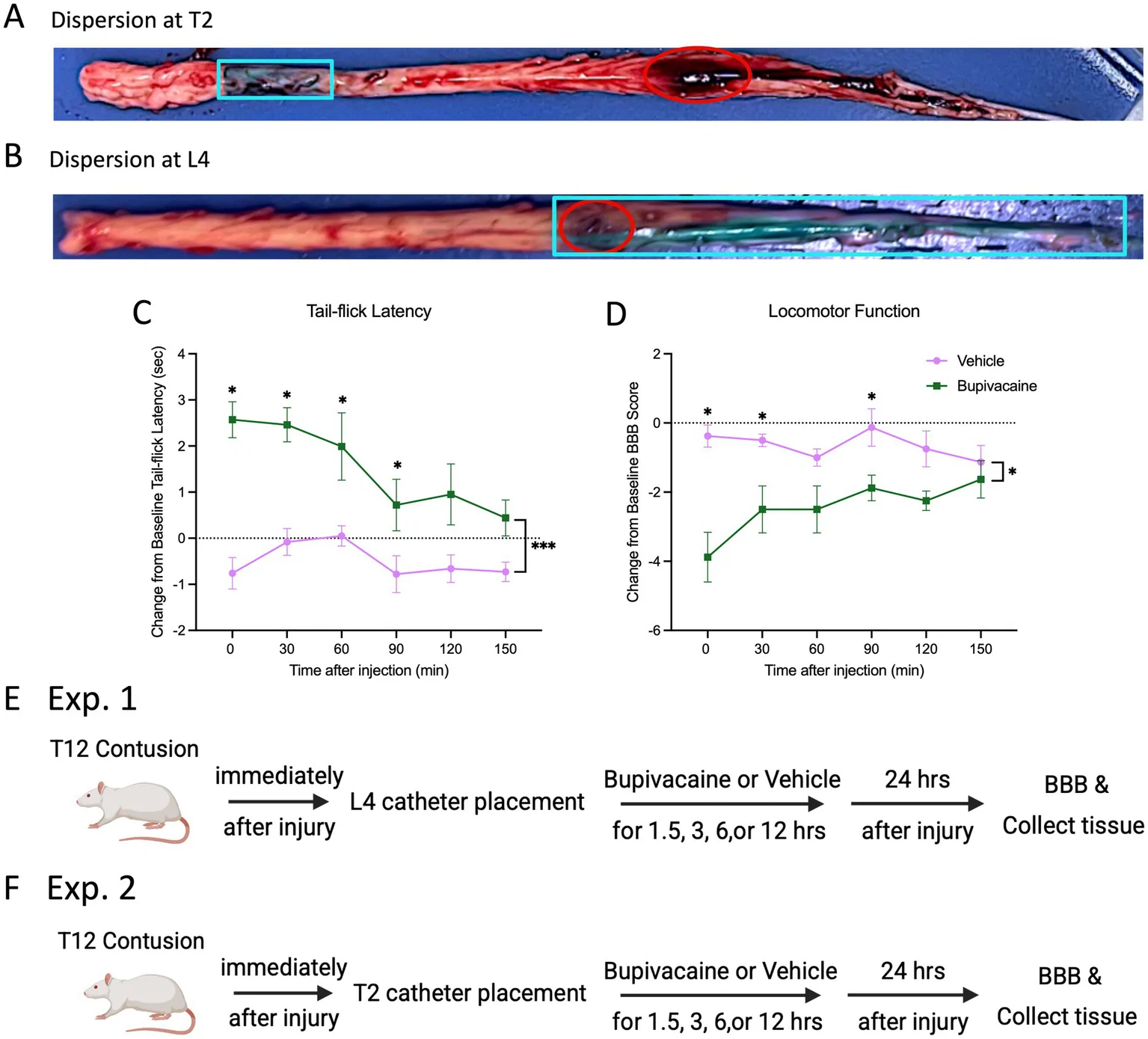
Experimental designs and fluid spread. **(A)** Fluid spread shown with blue dye infusion at T2 and **(B)** L4. Area encircled in red indicates the site of injury and the area boxed in blue indicates the extent of dye spread. Note that infusion at T2 is localized to the upper thoracic region, while infusion at L4 models a lumbar puncture and the dye reaches the site of injury. **(C)** Verification of the duration of spinal bupivacaine (green squares) on nerve blockade compared to vehicle controls (pink circles). **(D)** Verification of the duration of spinal bupivacaine on motor impairment. **(E)** Design for Experiment 1. **(F)** Design for Experiment 2. * indicates statistical significance (*p* < 0.05). *** (*p* < 0.005). Error bars represent standard error of the mean (*n* = 4). **E,F** were created in BioRender. Kobayashi, N (2026), https://BioRender.com/h12fv48.

From prior work, we knew that placing the catheter tip at T2 would lead to limited fluid dispersion restricted to the upper thoracic region of the spinal cord (12). As a preliminary verification, we slowly microinjected 30 μL of blue dye through a catheter with the tip positioned at T2, collected the spinal cord tissue, and assessed the extent of dye spread. As expected, the dispersion of fluid was limited to a small (approximately 1.0 cm) region surrounding T2 (Figure 1A). The L4 placement was selected to emulate an L5 lumbar puncture, which we anticipated would lead to a wider area of dispersion. Histology confirmed that dye applied at L4 dispersed across the lumbosacral region and extended to the site of injury (Figure 1B).

### 2.3 Drug administration

Rats were slowly infused with 30 μL of 0.75% bupivacaine (Pfizer, New York, NY) or 0.9% saline (vehicle), followed by a 10 μL saline flush through the implanted catheter. To extend the duration of treatment, additional drug infusions were given at 90 min intervals. Prior work suggested that bupivacaine produces a nerve blockade lasting 109 +/− 14 min in rats (16). A pilot experiment was conducted to verify that the drug remained active for at least 90 min. Bupivacaine was infused through a catheter positioned at L4 and motor/sensory function was assessed at 30 min intervals for 3 h. Locomotor function was evaluated using the Basso, Beattie, and Bresnahan (BBB) Locomotor Rating scale (17). Nociceptive sensory testing was performed by recording the latency at which rats exhibit a tail withdrawal in response to a noxious thermal stimulus (tail-flick test; IILC Life Science, Series 8 Model 33 T; Woodland Hills, CA). Spinal infusion of bupivacaine inhibited tail withdrawal (Figure 1C; *F*_(*1*,*6)*_ = 20.376, *p* < 0.005, *η^2^* = 0.460) and induced a motor impairment (Figure 1D; *F*_*(1*,*6)*_ = 6.655, *p* < 0.05, *η^2^ =* 0.382) for a period of 90 min, confirming this interval would be appropriate for the current study.

### 2.4 Behavioral testing

Over 3 separate days prior to experimentation, animals were acclimated to an open field for 4 min. Twenty-four hours after injury, locomotor function was assessed using the converted BBB scale, which has been shown to better conform to the assumptions of parametric tests compared to the original BBB scale (18). Hind paw movement in the hips, knees, and ankles was assessed by an observer blinded to treatment conditions over a period of 4 min.

### 2.5 Tissue collection

Animals were euthanized with 100 mg/kg of pentobarbital 24 h after injury. One cm of the spinal cord centered around the injury site was collected and flash frozen in liquid nitrogen. In addition, spleens were collected and weighed at the time of tissue collection.

Cord samples were stored at −80 °C. Protein was extracted from tissue samples and homogenized in QIAzol Lysis Reagent (QIAGEN, Hilden, Germany) according to manufacture directions. After removing RNA and DNA from the homogenized samples, the remaining protein extract underwent several rounds of purification with a guanidine-ethanol solution followed by centrifugation. The final protein pellet was left in the solution overnight and following drying the next day, a urea/DTT solution was used to break up and resolubilize the pellet. Roche cOmplete™ protease inhibitor (Sigma-Aldrich, St. Louis, MO) was added to prevent the protein from further degradation.

### 2.6 Spectrophotometry for hemorrhage

Spectral analyses for free hemoglobin were conducted on the extracted protein samples. Spectrophotometric absorbance (NanoDrop, Thermo Fisher Scientific, Waltham, MA) was measured from 200 to 800 nm for 1.5 μ L of each protein extract. Absorbance at 420 nm was used to assess oxygenated hemoglobin levels (19).

A Drabkin’s assay was conducted to calculate total hemoglobin content by quantifying cyanomethemoglobin levels. Twenty μL of protein extract was mixed with 160 μL of Drabkin’s Reagent (Sigma-Aldrich) and incubated at room temperature for 60 min. 1.5 μL of each mixture was loaded into a spectrophotometer to measure maximum absorbance of cyanmethemoglobin at 540 nm against a standard curve generated with known concentrations of bovine hemoglobin (Thermo Fisher Scientific).

### 2.7 Immunoblotting

To quantify the total protein concentration within each sample, a Bradford assay (Bio-Rad, Hercules, CA) was performed. Based on the assay results, protein samples were diluted in PBS, 4x XT buffer, and 20x XT reducing agent (Bio-Rad) to equal concentrations of 3 μg/μl.

These samples were analyzed for target proteins using SDS PAGE. Protein samples in sample buffer were heated at 96 °C for 5 min. Pre-cast 12% Bis-Tris gels (Bio-Rad) were placed into a Criterion cell and loaded with a standard XT MES running buffer (Bio-Rad). Ten μL of each protein sample was loaded into each well and 5 μL of Western C™ Blotting Standard (Bio-Rad) was added to the first and last well. Gels (2 per target per experiment) were run at 200 V for 45 min at 4 °C to prevent overheating during the run cycle. The gels were then equilibrated in 4 °C transfer buffer to prepare for transfer onto a methanol-activated polyvinylidene difluoride (PVDF, Millipore, Bedford, MA) membrane. The transfer apparatus was assembled according to the manufacturer’s instructions and run at 100 V for 1 h at 4 °C. Following transfer, the membranes were blocked with 5% Bovine Serum Albumin (VWR Chemicals, Solon, OH) in Tris-buffered saline with 0.1% Tween-20 (TBST) or 5% Non-Fat Dry Milk (ChemCruz, Dallas, TX) in 0.1% TBST for 1 h on a platform rocker. The blots were then incubated in primary antibody at 4 °C on a horizontal shaker overnight. The following primary antibodies were used: anti-hemoglobin-*α* [1:1000; Abcam (Cambridge, MA) ab92492, RRID: AB10561594], anti-caspase-3 [1:1000; Cell Signaling (Danvers, MA) 9,661 L, RRID: AB2341188], anti-interleukin-1β [1:1000; Abcam ab283818, RRID: AB3076536], anti-tumor necrosis factor (TNF)-α [1:1000; Abcam ab6671, RRID: AB305641], anti-immunoglobulin G [1:1000; Cell Signaling 70,775, RRID: AB10694715], anti-Iba-1 [1:1000; Abcam ab178846, RRID: AB2636859], anti-GFAP [1:5000; Abcam ab7260, RRID: AB305808], anti-GAPDH [1:20000; Proteintech 10,494-1-AP, RRID: AB2263076], or anti-lamin-B1 [1:2000; Proteintech 12,987-1-AP, RRID: AB2136290]. The following day, the membranes were washed 3 times with 0.1% TBST for 10 min each and incubated in horseradish peroxidase-conjugated goat anti-rabbit secondary antibody [1:10000; Abcam ab97051, RRID: 10679369] for 1 h at room temperature. After another 3 × 10 min series of washes in 0.1% TBST and a 5 min wash in Tris-buffered saline, the membranes were developed with an electrochemiluminescence substrate kit (Bio-Rad) and imaged with Fluorchem HD2 (ProteinSimple, Santa Clara, CA). Band intensity was measured using Bio-Rad Image Lab Software. Expression levels for each protein of interest were normalized to an appropriate housekeeping protein (Lamin-B1 or GAPDH), or ratioed to their conjugate form.

### 2.8 Statistics

The experiments employed factorial between-subjects designs, with 2 drug conditions (vehicle vs. bupivacaine) and 4 alternative treatment durations (1.5, 3, 6 or 12 h), yielding 8 independent groups. To evaluate the main effects of drug treatment and duration, and determine whether these factors interact, the parametric data were subject to an analysis of variance (ANOVA), which maintains the Type 1 error rate at 0.05. For our primary measures, we also report the proportion of variance accounted for (η^2^). In addition, we assessed group differences at each time point in Experiments 1 and 2 using a *t*-test and the Bonferroni correction (two-tailed). This correction divides the threshold for statistical significance (*p* < 0.05) by the number of comparisons (4), to minimize Type 1 errors. Thus, a *p* < 0.0125 was required to achieve statistical significance at a particular time point. A mediational analysis was performed using the jAMM module within jamovi. In this analysis, our primary factor [bupivacaine (drug) treatment] was related to the dependent variable protein concentration with hemorrhage (as measured by the Drabkin assay) as the mediator. Completely standardized effect sizes were estimated using the standard (delta) method. Similar results were obtained using the bootstrap (per-cent) method.

### 2.9 Experimental designs

#### 2.9.1 Experiment 1

We first examined whether targeting activity at the site of injury with bupivacaine has a protective effect against locomotor deficits, hemorrhage, and cell death/inflammation (Figure 1E). Immediately after a lower thoracic contusion, animals were implanted with a polyethylene catheter with the tip ending at the L4 level. Following this procedure, animals were randomly assigned to 4 treatment durations of 1.5, 3, 6, or 12 h and received either vehicle or bupivacaine through the catheter. This yielded a 2 (vehicle or bupivacaine) x 4 (treatment durations of 1.5, 3, 6, or 12 h) factorial design with six rats per group. Locomotor performance was assessed 24 h after the initial injury, which was followed by euthanasia and fresh tissue collection as described above.

#### 2.9.2 Experiment 2

Next, we examined whether applying bupivacaine rostral to injury (at T2) impacts hemorrhage, the expression of caspase 3, and locomotor function (Figure 1F). Immediately after a lower thoracic contusion, animals were implanted with a polyethylene catheter rostral to the site of injury with the tip ending at the T2 level. Following this procedure, animals were randomly assigned to 4 treatment durations of 1.5, 3, 6, or 12 h and received either vehicle or bupivacaine through the catheter. This yielded a 2 (vehicle or bupivacaine) x 4 (treatment durations of 1.5, 3, 6, or 12 h) factorial design with six rats per group. Locomotor performance was assessed 24 h after the initial injury, which was followed by euthanasia and fresh tissue collection as described above.

## 3 Results

To simplify the presentation of the results, we present the data from Experiments 1 and 2 together for each dependent variable. As the experiments were conducted at different times, separate ANOVAs were performed for each experiment.

### 3.1 Locomotor recovery

**Figure 2:**
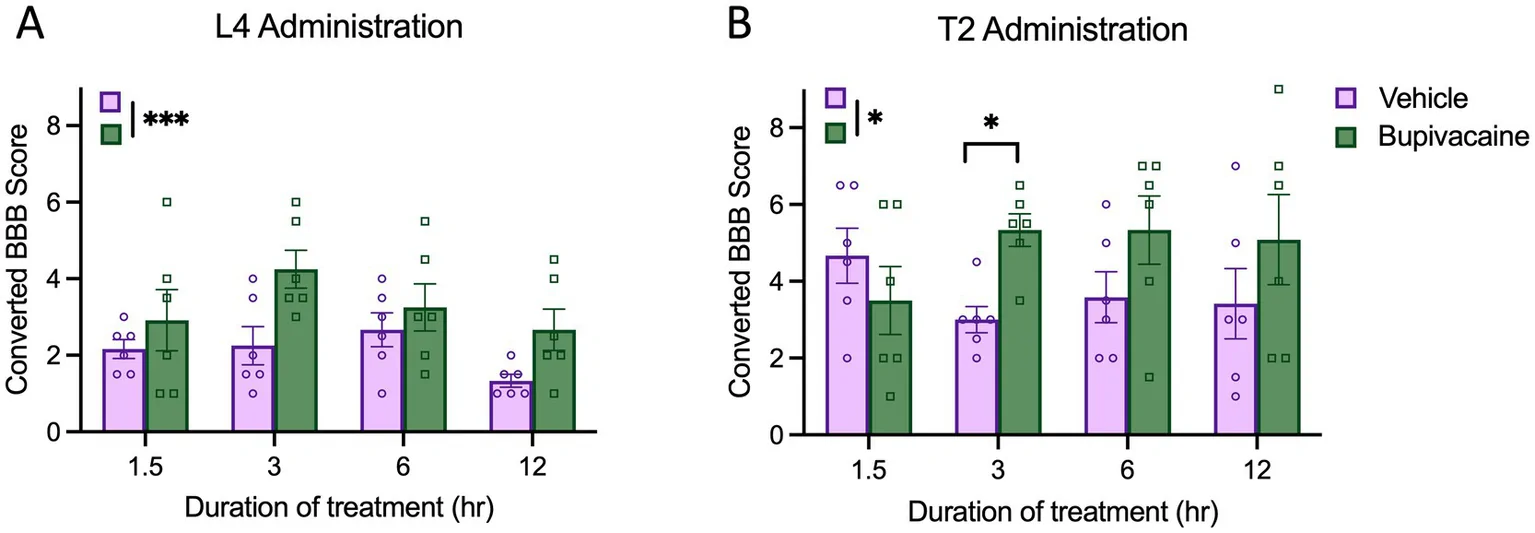
Bupivacaine anesthesia improved locomotor recovery after SCI. **(A)** L4 administration of bupivacaine improved locomotor recovery compared to vehicle-treated animals. **(B)** 3 h of bupivacaine administered at T2 improved locomotor function compared to animals that received vehicle infusions. Bupivacaine groups are indicated by green squares and vehicle by pink circles. * Indicates statistical significance (*p* < 0.05). *** (*p* < 0.005). Error bars represent standard error of the mean (*n* = 6).

Locomotor recovery was assessed using the converted BBB scale by a blind scorer as previously described. Bupivacaine-treated animals, particularly at the 3–12 h durations, showed improved locomotor recovery. For the L4 administration (Figure 2A), an ANOVA confirmed that drug treatment was statistically significant (*F*_*(*1, 40*)*_ *=* 10.426, *p <* 0.005, *η^2^ =* 0.175). No other term reached significance (both *F’s <* 2.256, *p >* 0.05). Similarly, the main effect of bupivacaine treatment was significant when the drug was given at T2 (*F*_*(*1, 40*)*_ *=* 4.170, *p <* 0.05, *η^2^ =* 0.083; Figure 2B). Here too, no other terms reached statistical significance (both *F’s <* 1.957, *p >* 0.05). Further analyses of the group means at each time point (using the Bonferroni correction) showed that the 3 h T2 bupivacaine-treated group differed from the 3 h control group (*p* < 0.0125).

### 3.2 Spleen weight

**Figure 3:**
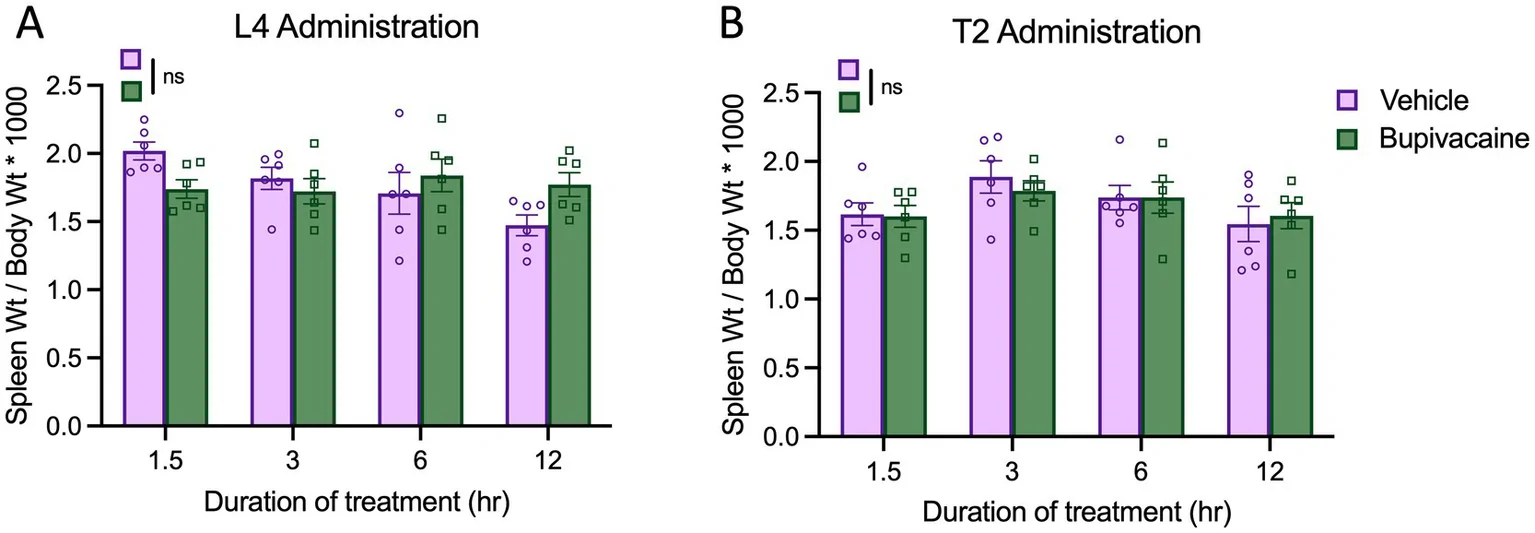
The effect of bupivacaine anesthesia on SCI-induced splenic atrophy. **(A)** L4 administration of bupivacaine blocked the splenic atrophy following SCI seen in the vehicle-treated group. **(B)** Time-dependent splenic atrophy was seen in both bupivacaine- and vehicle-treated animals when infused at the T2 level. Bupivacaine groups are indicated by green squares and vehicle by pink circles. Error bars represent standard error of the mean (*n* = 6).

Spleen weight normalized to the body weight (adjusted spleen weight) was assessed as a preliminary marker of immune dysregulation and showed a time-dependent effect of splenic atrophy. Spleen weight generally declined as the duration of treatment increased—an effect that was blocked by bupivacaine administered at L4, but not at T2. An ANOVA performed on the data from animals treated at L4 (Figure 3A) confirmed that the change in spleen weight over time depended upon drug treatment (*F*_*(*3, 40*)*_ *=* 3.434, *p <* 0.05, *η^2^ =* 0.179). The main effects of drug and duration were not significant (both *F’s <* 2.366, *p >* 0.05). For the rostral administration (Figure 3B), spleen weight varied with treatment duration (*F*_*(*3, 40*)*_ = 2.972, *p <* 0.05, *η^2^ =* 0.180), but was not affected by drug treatment (*F <* 1.0, *p >* 0.05).

### 3.3 Protein levels

**Figure 4:**
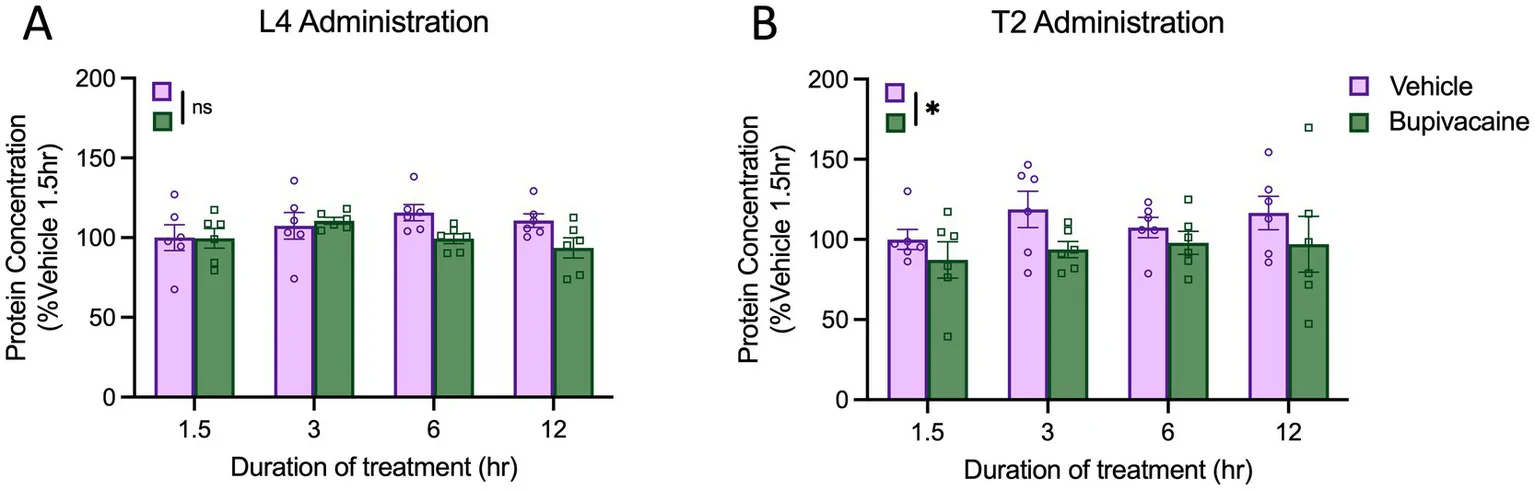
Bupivacaine anesthesia decreased overall protein concentration at the site of injury. **(A)** L4 administration of bupivacaine had no effect on protein concentration. **(B)** T2 administration of bupivacaine reduced protein concentration. Bupivacaine groups are indicated by green squares and vehicle by pink circles. * Indicates statistical significance (*p* < 0.05). Error bars represent standard error of the mean (*n* = 6).

The total protein concentrations within the injured neural tissue were measured using the Bradford assay and values are expressed as a percentage of the mean of the 1.5 h vehicle-treated group. Bupivacaine treatment generally reduced net protein content at the site of injury. An ANOVA performed on the data from animals given bupivacaine at L4 (Figure 4A) revealed a main effect of drug treatment that approached significance (*F*_*(*1, 40*)*_ *=* 3.498, *p =* 0.069, *η^2^ =* 0.067). Neither the main effect of duration, nor its interaction with drug treatment, approached significance (both *F’s <* 1.651, *p >* 0.05). An ANOVA performed on the data from animals given bupivacaine at T2 (Figure 4B) found a significant effect of drug treatment (*F*_*(*1, 40*)*_ *=* 5.394, *p <* 0.05, *η^2^ =* 0.112). Again, the main effect of duration and its interaction with drug treatment were not significant (both *F’s <* 1.0, *p >* 0.05).

### 3.4 Markers of hemorrhage

Levels of hemorrhage at the site of injury were assessed using three different assays: absorbance at 420 nm (NanoDrop), Drabkin’s assay for cyanmethemoglobin, and Western blotting for *α*-hemoglobin. Across the three assays, bupivacaine applied at T2 reduced hemorrhage. Drug application at L4 had a more modest effect that was only evident with the Drabkin’s assay.

**Figure 5:**
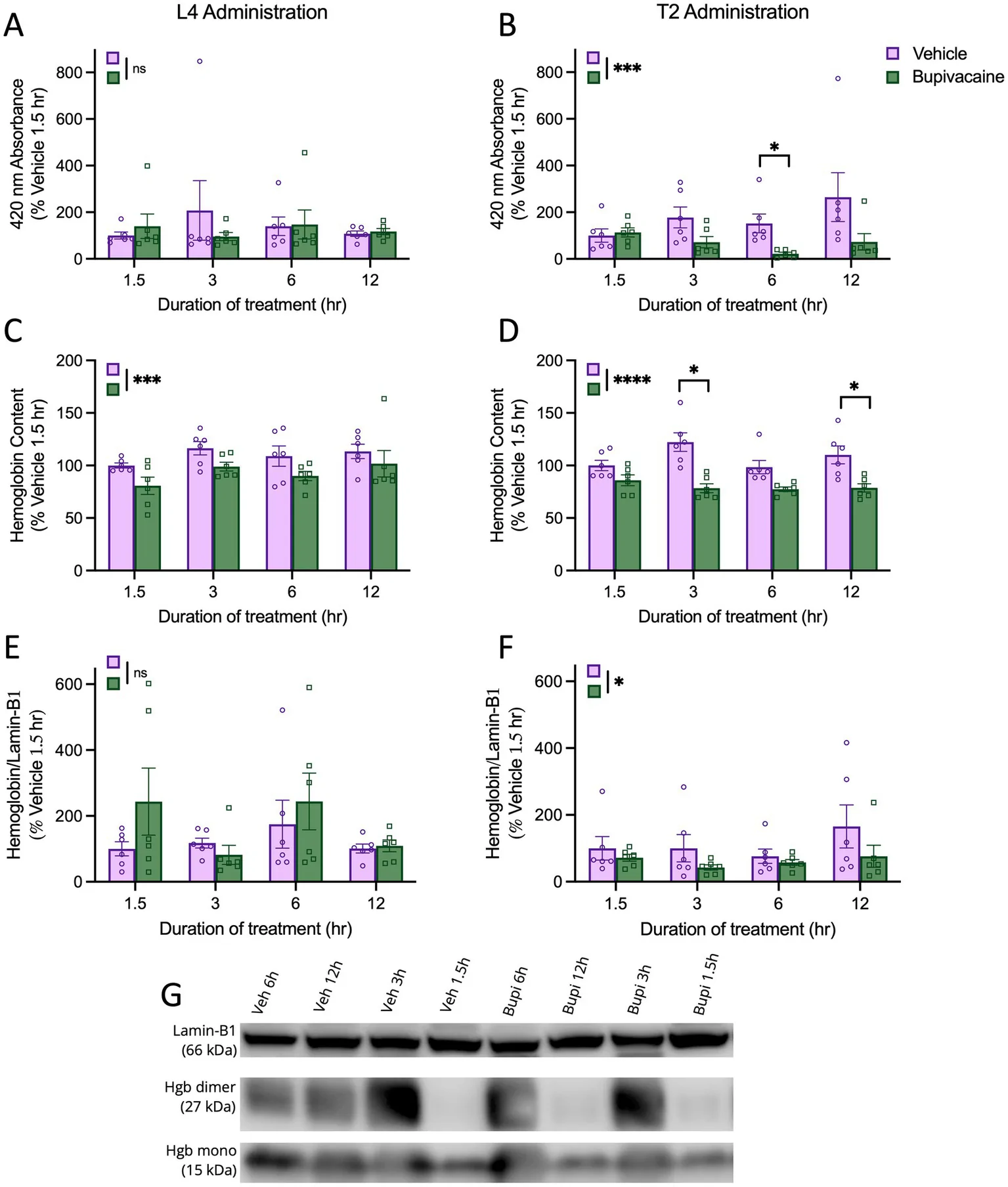
Bupivacaine anesthesia decreased markers of hemorrhage at the site of injury. **(A)** L4 administration of bupivacaine had no effects on free hemoglobin content as measured by light absorbance at 420 nm. **(B)** T2 administration of bupivacaine decreased absorbance at 420 nm when continued for 6 h. **(C)** Hemoglobin content measured with the Drabkin’s assay was reduced in L4 bupivacaine-treated animals when compared to the vehicle-treated animals. **(D)** Similarly, T2 administration of bupivacaine reduced hemoglobin content, particularly at 3 and 12 h durations. **(E)** Western blotting of *α*-hemoglobin normalized to lamin-B1 failed to show any group differences when treatment was applied at L4. **(F)** Expression of α-hemoglobin was lowered when bupivacaine was administered to target T2. **(G)** A representative immunoblot revealed reduced labeling of hemoglobin at 27 kDa (dimer) and 15 kDa (monomer) in samples from T2 bupivacaine-treated rats. Bupivacaine groups are indicated by green squares and vehicle by pink circles. * Indicates statistical significance (*p* < 0.05), *** (*p* < 0.005), **** (*p* < 0.0001). Error bars represent standard error of the mean (*n* = 6).

Prior work has shown that samples from animals with hemorrhage have a red tint, which can be assessed by measuring absorbance at 420 nm, the wavelength associated with hemoglobin (19). Here, and in subsequent assays, we normalized the data from each experiment, with the data from the 1.5 h vehicle-treated group as the benchmark (set to 100%). Bupivacaine had no effect on absorbance at 420 nm when applied at L4 (Figure 5A), but reduced absorbance when applied at T2 for 3 or more hours (Figure 5B). An ANOVA performed on the data from L4 treated animals confirmed that neither the main effect of drug nor duration of treatment were statistically significant (both *F’s <* 1.0, *p >* 0.05). For T2-treated rats, there was a significant effect of drug treatment (*F*_*(*1, 40*)*_ *=* 9.779, *p <* 0.005, *η^2^ =* 0.168), but not duration (*F =* 1.105, *p >* 0.05). Comparisons of the group means at each time point revealed a significant difference for 6 h T2 treated animals (*p* < 0.0125).

A secondary measure of hemoglobin content was obtained using the Drabkin’s assay. With this assay, bupivacaine applied at L4 (Figure 5C) and T2 (Figure 5D) reduced hemorrhage. An ANOVA performed on the data from L4 treated rats revealed a significant effect of drug treatment (*F*_*(*1, 40*)*_ *=* 10.131, *p <* 0.005, *η^2^ =* 0.176) and an effect of treatment duration that approached significance (*F*_*(*3, 40*)*_ *=* 2.412, *p =* 0.0810, *η^2^ =* 0.125). For animals treated at T2, there was a significant effect of drug treatment (*F*_*(*1, 40*)*_ *=* 43.349, *p <* 0.0001, *η^2^ =* 0.456). For this condition, the main effect of duration was not significant (*F =* 1.479, *p >* 0.05), but the interaction between duration and drug treatment approached significance (*F*_*(*3, 40*)*_ *=* 2.397, *p =* 0.0823, *η^2^ =* 0.075). Additional analyses of the group differences at each time point revealed a significant effect of T2-administered bupivacaine at 3 and 12 h (*p* < 0.0125).

Lastly, *α*-hemoglobin within the injured tissue was measured using Western blotting and normalized to a housekeeping protein, lamin-B1. To confirm that differences in housekeeping protein expression were not skewing our data, an ANOVA was performed, revealing no significant differences between drug treatment or duration on lamin-B1 levels for both Experiments 1 and 2 (all *F’s* < 2.431, *p* > 0.05). Western blotting for hemoglobin showed that the drug was effective when administered at T2 (Figures 5F,G), but not L4 (Figure 5E). An ANOVA verified that neither the effect of drug treatment nor duration were significant when the drug was administered at L4 (both *F’s <* 1.803, *p >* 0.05). An ANOVA performed on the data from T2 treated animals found a significant effect of drug treatment (*F*_(1, 40)_ = 4.222, *p <* 0.05, *η^2^ =* 0.086), but not duration (*F =* 1.072, *p >* 0.05).

### 3.5 Markers for cell death and inflammation

**Figure 6:**
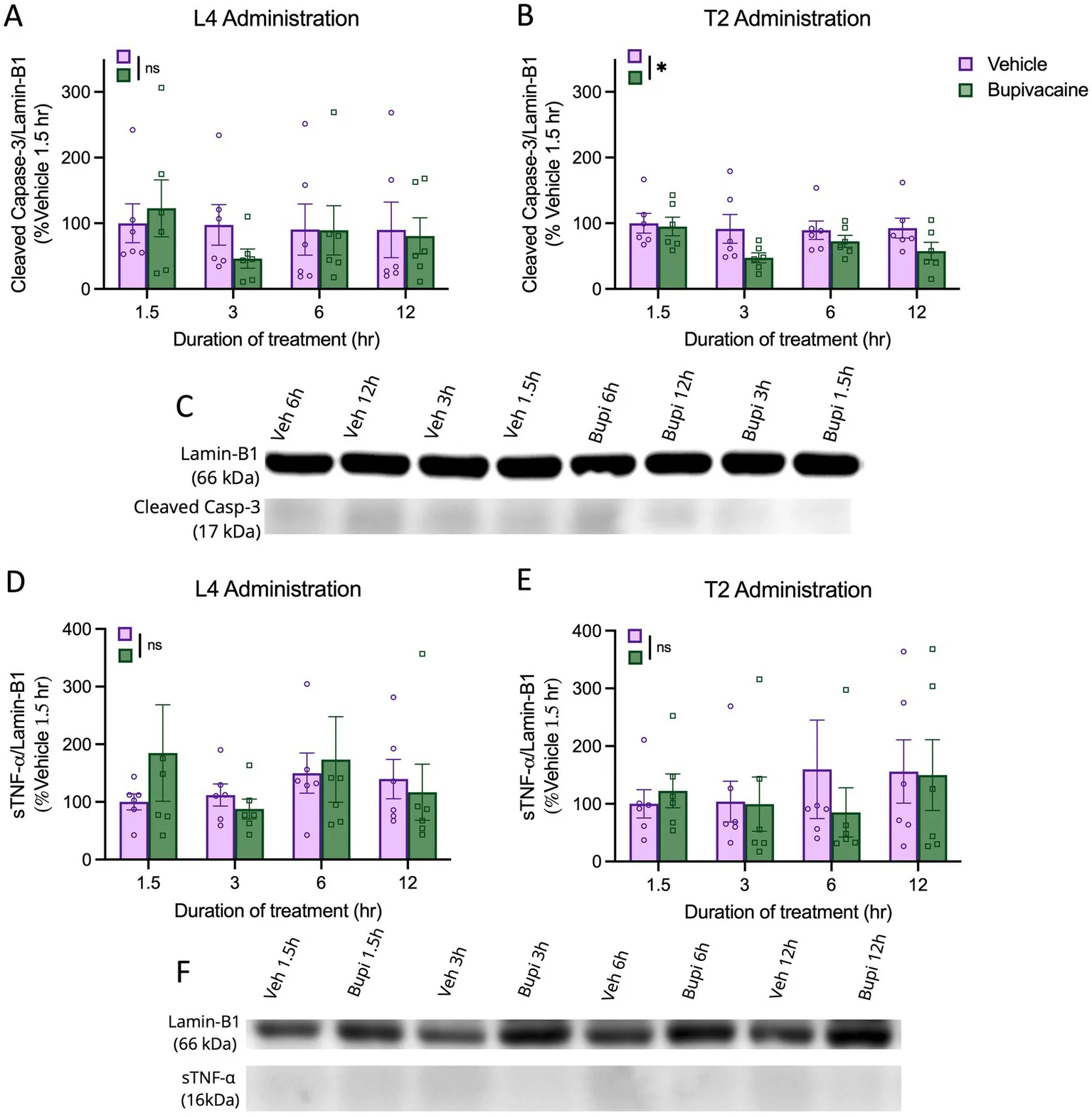
Bupivacaine anesthesia had a site dependent effect on the expression for cell death, but not inflammation. **(A)** Expression levels of caspase-3, a marker of cell death, did not change between the drug treatments when infused at the L4 level. **(B)** T2 administration of bupivacaine decreased levels of caspase-3 compared to the vehicle-treated animals. **(C)** A representative immunoblot revealed reduced labeling at 17 kDa (cleaved caspase-3) in samples from T2 bupivacaine treated rats. **(D,E)** Neither L4 or T2 administration of bupivacaine resulted in differences on TNF expression, a pro-inflammatory cytokine. **(F)** A representative immunoblot revealed no differences in labeling at 16 kDa (soluble TNF-α) in samples from T2 bupivacaine treated rats. Bupivacaine groups are indicated by green squares and vehicle by pink circles. * Indicates statistical significance (*p* < 0.05). Error bars represent standard error of the mean (*n* = 6).

Relative levels of cell death and inflammation were assessed by probing for the cleaved form of caspase-3 and the soluble form of tumor necrosis factor (sTNF-*α*) using Western blotting. Both markers are expressed as values normalized to the housekeeping protein, lamin-B1. Caspase-3 expression was reduced by bupivacaine applied at T2 (Figures 6B,C), but not at L4 (Figure 6A). An ANOVA showed that neither the effect of drug treatment nor duration was significant for the L4 condition (both *F’s <* 1.0, *p >* 0.05). For the T2 administration group, there was a significant effect of drug treatment (*F*_*(*1, 40*)*_ *=* 6.171, *p <* 0.05, *η^2^ =* 0.117), but not duration (*F =* 1.418, *p >* 0.05). Neither L4 (Figure 6D) nor T2 (Figures 6E,F) bupivacaine treatment affected sTNF-α expression (all *F’s <* 1.0, *p* > 0.05).

**Figure 7:**
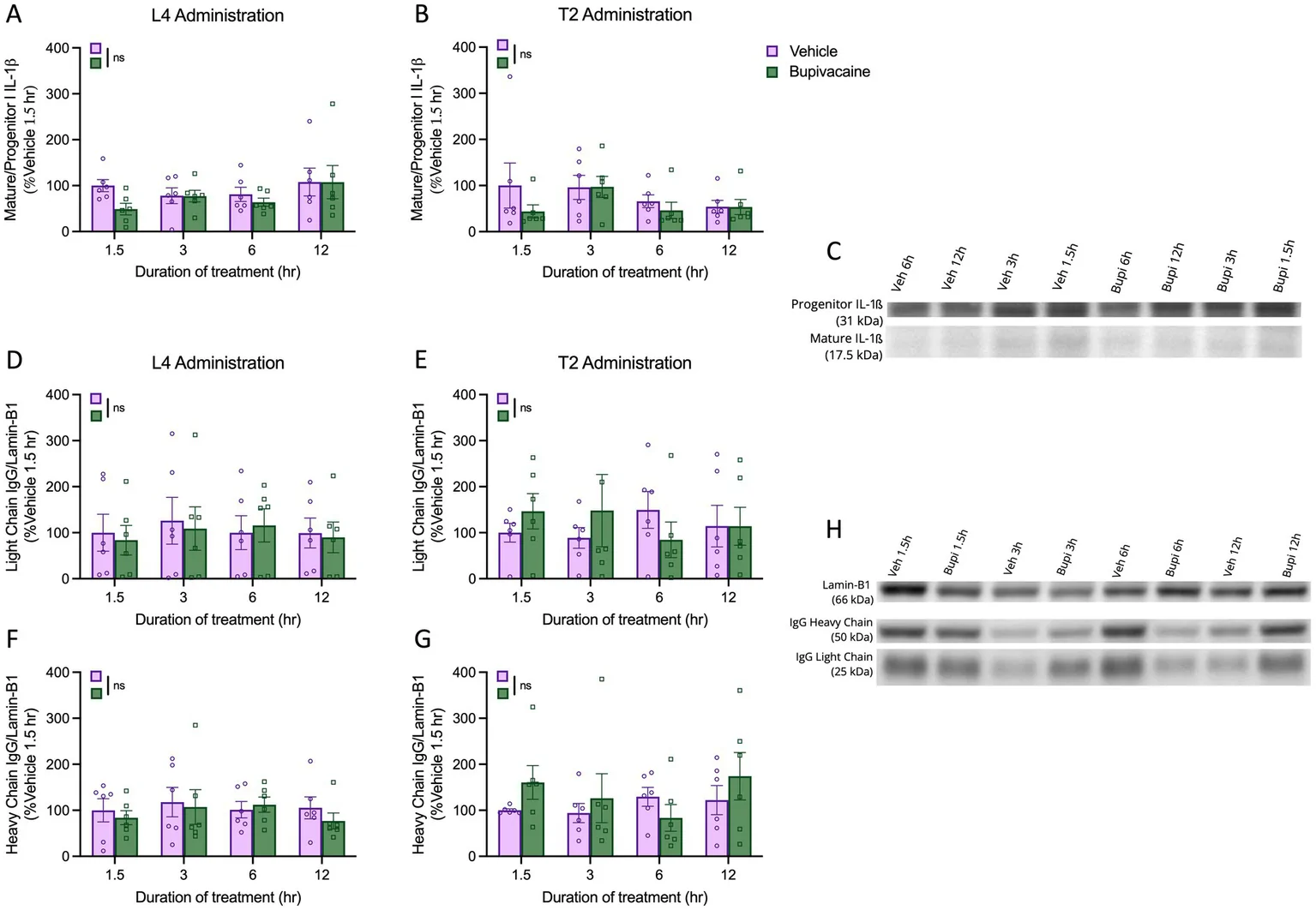
Bupivacaine anesthesia did not affect expression of IL-1β and IgG. **(A,B)** Neither L4 nor T2 administration of bupivacaine resulted in group differences on IL-1β expression, a marker of inflammation. **(C)** A representative immunoblot revealed no differences in labeling at 31 kDa (progenitor IL-1β) and 17.5 kDa (mature IL-1β) in samples from T2 bupivacaine-treated rats. **(D–G)** Neither L4 nor T2 administration of bupivacaine resulted in differences on light chain and heavy chain IgG expression. **(H)** A representative immunoblot revealed no differences in labeling at 50 kDa (heavy chain IgG) and 25 kDa (light chain IgG) in samples from T2 bupivacaine-treated rats. Bupivacaine groups are indicated by green squares and vehicle by pink circles. Error bars represent standard error of the mean (*n = 6*).

We also measured interleukin (IL)-1ß and immunoglobulin G (IgG) expression by Western blotting to assess the extent of the immune response. The ratio of mature to progenitor forms of IL-1ß are expressed as a percentage relative to 1.5 h vehicle-treated animals. Light- and heavy-chain forms of IgG normalized to lamin-B1 were analyzed separately. Bupivacaine treatment had no effect on the expression of these markers. An ANOVA confirmed that for both the L4 (Figure 7A) and T2 (Figures 7B,C) conditions, neither drug treatment nor duration had a significant effect on IL-1ß expression (all *F’s <* 1.442, *p* > 0.05). Similarly, no significant differences in drug treatment or duration were found on IgG expression in both L4 (Figures 7D,F) and T2 (Figures 7E,G,H) conditions (all *F*’s < 1.024, *p >* 0.05).

**Figure 8:**
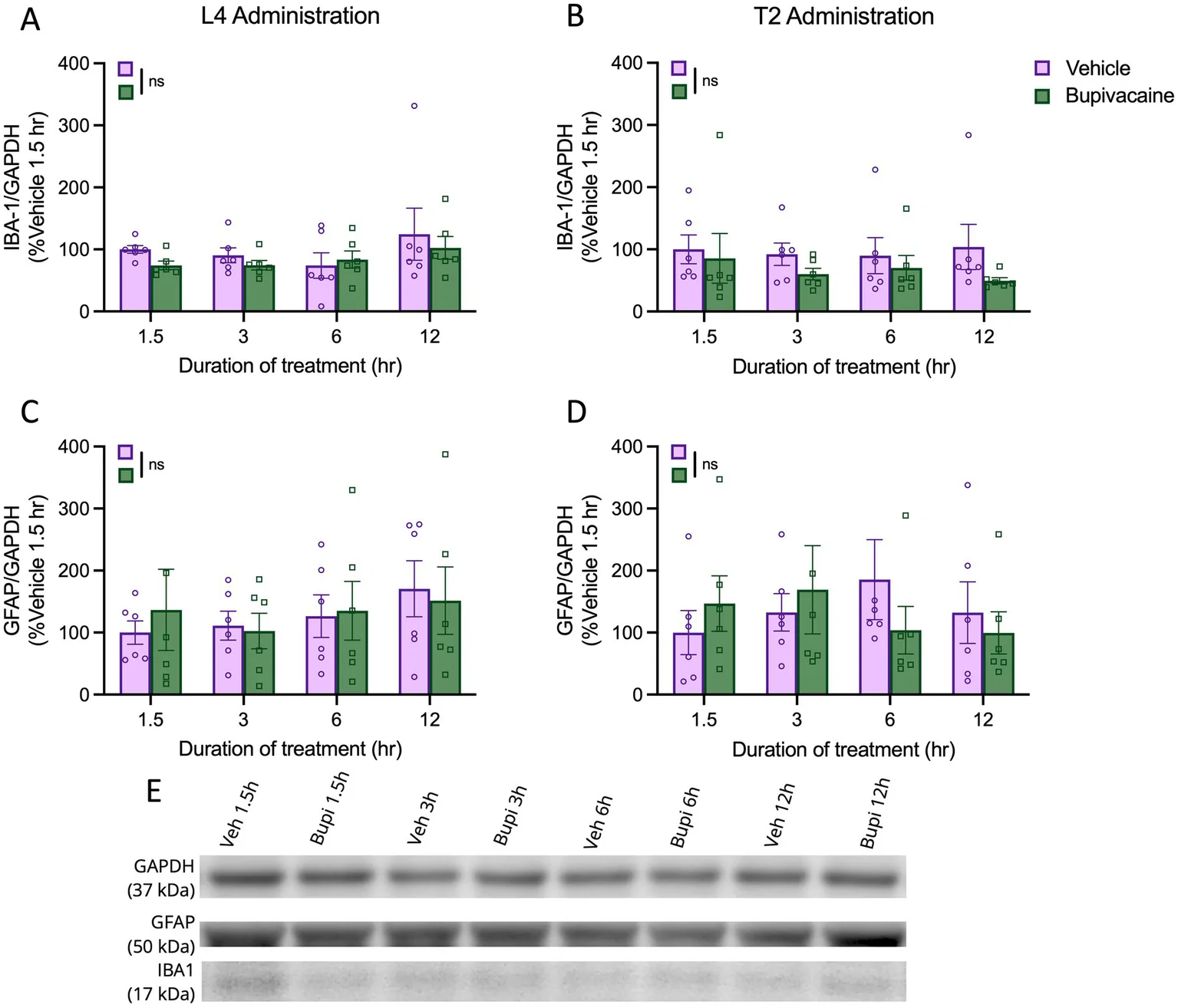
Bupivacaine anesthesia did not affect expression of Iba-1 or GFAP. **(A,B)** Expression of Iba-1, a marker of microglia activation, was not affected by L4 or T2 administration of bupivacaine. **(C,D)** Neither L4 nor T2 administration of bupivacaine resulted in differences of GFAP expression, a marker of astrocyte activation. **(E)** A representative immunoblot revealed no differences in labeling at 50 kDa (GFAP) and 17 kDa (Iba-1) in samples from T2 bupivacaine-treated rats. Bupivacaine groups are indicated by green squares and vehicle by pink circles. Error bars represent standard error of the mean (*n* = 6).

To examine levels of active microglia and astrocytes, Western blotting was performed to probe for Iba-1 and GFAP, respectively. Both markers are shown as values normalized to a loading control, GAPDH. Bupivacaine treatment had no effect on the expression of Iba-1. An ANOVA revealed that for the L4 (Figure 8A) and T2 (Figures 8B,E) conditions, neither drug treatment nor duration had a significant effect on Iba-1 expression (all *F’s <* 2.846, *p* > 0.05). Similar, no significant differences in drug treatment or duration were found on GFAP expression in both L4 (Figure 8C) and T2 (Figures 8D,E) conditions (all *F’s <* 1.0, *p* > 0.05).

### 3.6 Comparisons and summary of findings

**Figure 9:**
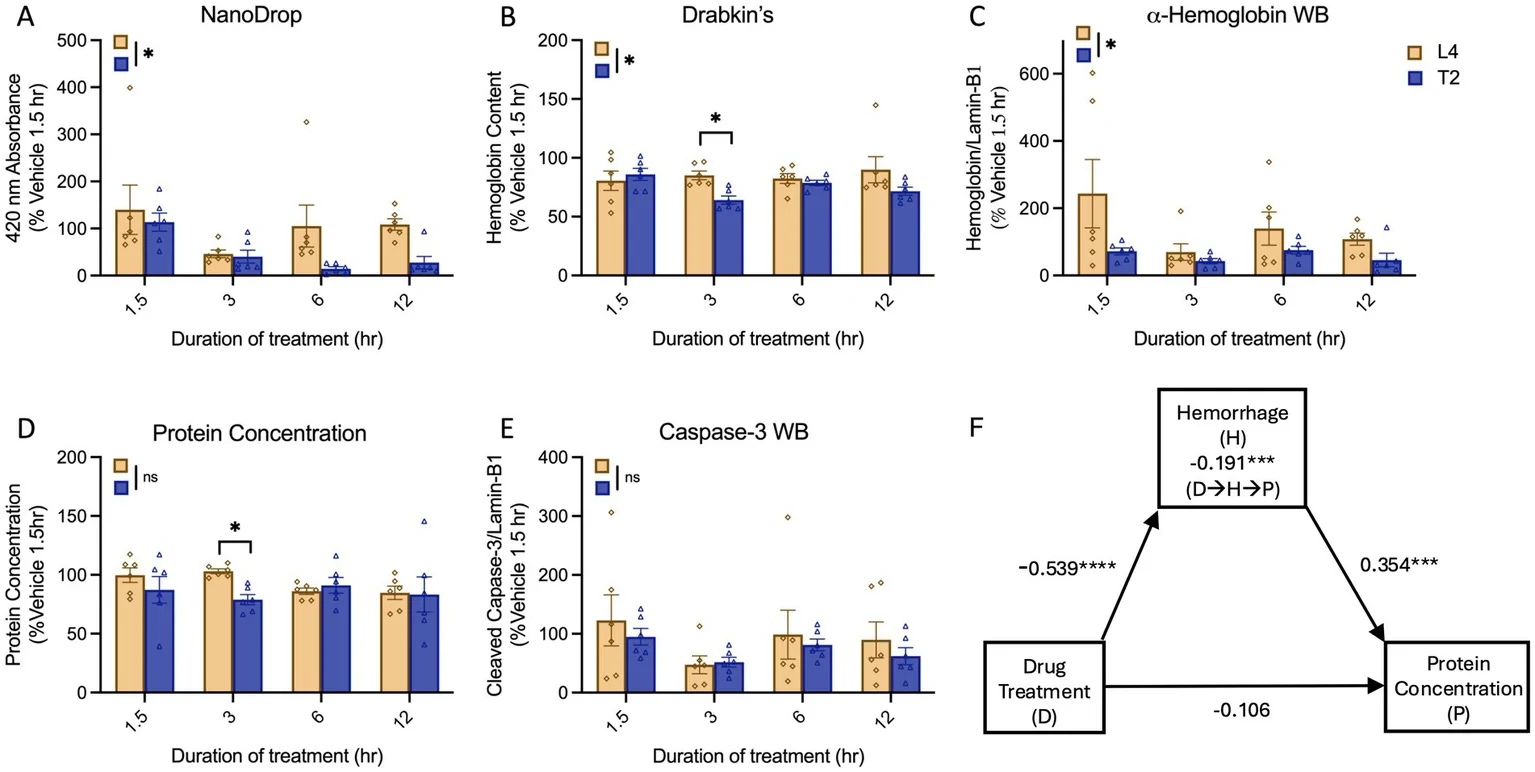
Summary graphs of significant findings and comparisons between site of administration in bupivacaine treated animals. **(A)** T2 administration (blue triangles) of bupivacaine reduced absorbance at 420 nm compared to L4 administration (yellow diamonds). **(B)** Reduced levels of hemoglobin content were seen in groups administered bupivacaine at T2 compared to L4. **(C)** Bupivacaine administered at T2 yielded lower α-hemoglobin expression, as measured by Western blotting (WB), compared to L4 administration. **(D)** Protein concentration differed only at 3 h of bupivacaine administration between T2 and L4. **(E)** No group differences were found in expression levels of cleaved caspase-3. **(F)** A mediational analysis showed that drug treatment (D) reduced hemorrhage (H). An increase in hemorrhage (H) predicted a rise in protein (P). The increase in protein concentration was strongly predicted by an increase in hemorrhage, but not the direct effect of drug treatment. The values along each line indicate the standardized effect sizes (beta values). The indirect effect is indicated below the label set in parentheses, indicating the path. * Indicates statistical significance (*p* < 0.05), *** (*p* < 0.005), **** (*p* < 0.0001). Error bars represent standard error of the mean (*n* = 6).

Our cellular assays suggest that bupivacaine administered rostral to injury had a more robust effect. To further explore this outcome, additional comparisons were performed on the normalized data from Experiments 1 and 2. For these L4/T2 comparisons, we focused on cellular assays that produced a significant effect of drug treatment. First, we compared the vehicle control groups. For these animals, neither the main effect of infusion site, nor its interaction with duration, was statistically significant (all *F*’s *<* 1.315, *p* > 0.05). To assess the relative effectiveness of bupivacaine at each time point, we computed the percentage change from the vehicle-treated animals by dividing the data from bupivacaine-treated animals at each time point by the average value of the vehicle control group at the same time point. On this scale, scores less than 100% indicate reduced expression in bupivacaine-treated animals. This conversion allowed us to compare the data from bupivacaine-treated animals across infusion site using statistical analyses identical to those described above. These analyses showed that bupivacaine reduced hemorrhage more when given at T2 (Figures 9A–C). Separate ANOVAs confirmed that there was an effect of infusion site on absorbance at 420 nm (*F*_*(*1, 40*)*_ *=* 7.283, *p <* 0.05, *η^2^ =* 0.117), the Drabkin’s assay (*F*_*(*1, 40*)*_ *=* 5.113, *p <* 0.05, *η^2^ =* 0.094), and Western blotting for *α*-hemoglobin (*F*_*(*1, 40*)*_ *=* 7.305, *p <* 0.05, *η^2^ =* 0.128). While the overall difference for protein concentration (Figure 9D) was not significant (*F*_*(*1, 40*)*_ = 2.124, *p >* 0.05), comparisons at each time point revealed a significant difference for 3 h of treatment (*p* < 0.0125). Bupivacaine at T2 produced a greater reduction in cleaved caspase-3 expression (Figure 9E), but this effect was not statistically significant (*F*_*(*1, 40*)*_ = 1.867, *p >* 0.05).

In the present study, we found that a treatment (bupivacaine) that lowered hemorrhage also reduced protein content in our samples. A converse effect has been observed in studies of polytrauma, where we have found that noxious stimulation increases hemorrhage and protein concentration. In both cases, we assume that the change in protein concentration stems from the infiltration of hemoglobin. To further explore this issue, we performed an additional analysis to test whether the effect of drug treatment on hemorrhage (as measured by the Drabkin’s assay) mediates its effect on protein concentration (Figure 9F). This analysis confirmed that bupivacaine treatment impacted hemorrhage (z = -6.270, *p* < 0.0001). Further, the rise in protein concentration was related to increased hemorrhage (z = 3.224, *p* = 0.0013). Most importantly, the indirect effect of bupivacaine treatment, mediated by hemorrhage, predicted the change in protein concentration (z = -2.867, *p* = 0.0041). Excluding this mediator, there was no (direct) effect of drug treatment on protein concentration (z = -0.962, *p* > 0.05). Comparable analyses examining the rise in protein concentration produced by noxious stimulation reveal an analogous effect, showing that this is mediated by nociception-induced hemorrhage (20).

## 4 Discussion

### 4.1 Overview

The current experiments explored whether regional anesthesia has a protective effect, in the absence of noxious stimulation, if initiated soon after injury. A longer-lasting anesthetic (bupivacaine) was applied via a catheter with the tip positioned caudal (at L4) or rostral (at T2) to a T12 contusion injury. As previously reported (12), infusion at T2 led to limited dispersion. More widespread dispersion, extending from the area of injury to the lower lumbosacral spinal cord, was observed when the infusion was made at L4. We then evaluated the effect of applying bupivacaine 1, 2, 4, or 8 times at 90 min intervals at T2 or L4, with the first infusion occurring within 5 min of injury. This yielded four treatment durations: 1.5, 3, 6, or 12 h. A pilot experiment showed that bupivacaine inhibited motor and sensory function for a period of 90 min. We predicted that L4 bupivacaine would have a protective effect and that the magnitude of this effect would grow with treatment duration. In the absence of additional nociceptive stimulation, there was little reason to expect a role of rostral systems, and for this reason, we hypothesized that rostral bupivacaine would have minimal effect.

Behavioral function was assessed a day after injury and tissue was collected immediately after. At this time point, a minimum of 13.5 h after the last drug infusion [for animals that received 8 infusions (12 h condition)], there was no evidence of a bupivacaine-induced disruption in motor function. Instead, animals that received bupivacaine infusions for 3–12 h exhibited improved motor performance. Further, a quantitative measure of hemoglobin at the site of injury (Drabkin’s assay) suggested that regional anesthesia attenuated hemorrhage. Bupivacaine infused at L4 did not, however, have a significant effect on the expression of caspase-3, IL-1ß, TNF-*α*, IgG, Iba-1, or GFAP. Interestingly, repeated vehicle infusions at L4 led to a drop in spleen weight, which suggests that the infusion procedure engaged systemic processes, potentially linked to cumulative stress. This duration-dependent splenic atrophy was blocked by bupivacaine applied at L4.

Contrary to our initial hypothesis, bupivacaine had a robust protective effect when applied rostral to injury. Infusing bupivacaine at T2 for 3–12 h attenuated the loss in locomotor function, increase in hemorrhage (across three assays), and the expression of caspase-3, but had no effect on indices of inflammation (IL-1ß, TNF-α, IgG, Iba-1, or GFAP). Caspase-3 is known to initiate apoptosis through DNA fragmentation and degradation of cell integrity (21). Disruption of the BSCB after SCI can lead to infiltration of neurotoxic components within blood, such as hemoglobin or free iron, into neural tissue, which can trigger caspase-3 activation, leading to cell death (22, 23). Supporting this hypothesis, others have reported that bupivacaine rescues BSCB integrity in a peripheral inflammatory pain model (24). We also found that bupivacaine reduced protein content at the site of injury. A mediation analysis suggested that the extent of hemorrhage predicted (mediated) the increase in protein concentration, implying that this reflects the infiltration of blood-borne contents.

We hypothesized that the protective effect of bupivacaine would increase with the duration of treatment. When applied at T2, the drug was effective when applied for minimum of 3 h, but not with a single administration (1.5 h condition). This pattern of results could emerge because the first 90 min of treatment has little effect (possibly because cellular activity is muted by spinal shock) or because a positive effect requires continued treatment for 180 min (two infusions). Further work is needed to determine whether delayed drug administration after injury has an effect. This is particularly important for clinical application because early treatment (within the first 90 min of injury) is typically not possible.

While we hypothesized that increasing the duration of treatment would bring additional benefit, there was little indication that bupivacaine applied at T2 for more than 3 h (an additional 4–8 infusions at 90 min intervals) brought further benefit. The implication is that key brain-dependent processes are enlisted within 3 h and that blocking their engagement at this time can have a lasting protective effect.

### 4.2 Comparison of the effect of bupivacaine to nociception-induced tissue loss

The current results stand in contrast to our prior work examining the effect of regional anesthesia administered a day after injury, where no effect was observed in the absence of nociceptive stimulation. The present findings show that bupivacaine applied soon after injury can have a protective effect that reduces hemorrhage, cell death, and the acute loss of locomotor function. In contrast to what is observed with noxious stimulation, where lidocaine attenuates indices of inflammation (10), regional anesthesia had no effect on inflammation when applied soon after injury. This may be attributed to the acuteness of the current study. Many inflammatory markers rise after 12–24 h, with the onset of reparative and degenerative processes (25). In the current study, bupivacaine was administered within the first 12 h and, for this reason, may have had less effect on the inflammatory response. Likewise, variation in inflammation related to cell death may lag the expression of caspase-3. Further studies will be necessary to examine whether early anesthesia affects how inflammatory processes unfold over time.

The results also diverge with regard to the relative effectiveness of drug infusion at L4 versus T2. When given prior to noxious stimulation, regional anesthesia at both sites has a similar effect (10, 12), whereas anesthesia at T2 appears more effective when treatment is initiated soon after injury. The latter observation suggests that the protective effect of regional anesthesia may be partly due to the inhibition of ascending/descending fibers rostral to injury, which parallels research implicating brain systems in pain-induced hemorrhage. Additional parallels are suggested by studies examining the effect of placing animals in a coma-like state using pentobarbital, which blocks nociception-induced hemorrhage a day after injury (13). Likewise, maintaining a state of general anesthesia for 12 h after injury reduces hemorrhage at the site of injury (20). The fact that rostral bupivacaine had a more robust effect also speaks to an alternative interpretation of the finding—that the drug was effective because it diffused caudal to the injured cord. If this was the case, caudal bupivacaine treatment would have been more effective, but the opposite was observed.

Elsewhere, we have hypothesized that regional anesthesia at T2 impacts nociception-induced tissue loss and hemorrhage because it inhibits activity in surviving afferent fibers and the engagement of brain systems (3). Additionally, neural overexcitation/inflammation at the injury site could have a systemic effect that is detected by brain systems. In the absence of noxious stimulation, there are a number of factors that could influence brain systems after injury. SCI in itself is known to produce a depolarizing shift in GABA at, and caudal to, the site of injury (26, 27), which could enable a state of over-excitation that drives afferent activity after injury. Recent work suggests that countering this shift in GABA function has a protective effect (28, 29). We also acknowledge that tissue damage related to performing the contusion injury could provide a source of afferent drive and systemic stress that may engage brain systems.

Other work suggests that the engagement of a sympathetic response contributes to pain-induced tissue loss after SCI. Noxious stimulation elicits a rise in blood pressure and blood flow that could foster the breakdown of the BSCB via capillary fragmentation, enabling hemorrhage and cell death at the site of injury (30). This is consistent with recent studies showing that bouts of hypertension after injury are associated with poor recovery (31, 32). For polytrauma, inducing a state of general anesthesia with pentobarbital attenuates both the pain-induced rise in blood pressure and hemorrhage (13). Similar results are obtained when general anesthesia is maintained for 12 h after injury—pentobarbital lowered blood pressure and hemorrhage (20). Further, if autonomic activity and cardiovascular drive contribute to tissue loss after injury, this could explain why bupivacaine has a robust effect when applied in the upper thoracic (T2) region, where preganglionic nerves that drive the sympathetic rise in blood pressure and heart rate exit the spinal cord (33, 34). Importantly, local anesthetics are known to be preferentially sensitive to (B) fibers that mediate sympathetic activity (15). Further work is needed to elucidate why regional anesthesia at T2 has a protective effect.

Additionally, we need to consider the role of fibers that project from the brain to the spinal cord. Prior studies suggest that neural excitability within the spinal cord central gray is modulated by axons that descend through the dorsolateral funiculi (35, 36). Recent evidence suggests that cutting this pathway rostral to a lower thoracic contusion injury attenuates the adverse effects of noxious stimulation on locomotor function and hemorrhage (37). Further work is needed to determine whether these pathways contribute to injury-related pathophysiology in the hours after SCI.

Finally, recent studies show that SCI drives a reduction in spleen weight that is correlated with an increase in splenic inflammatory cytokine profile (38, 39). In the present study, repeated drug administration led to spleen atrophy. While this effect was attenuated by bupivacaine applied at L4, the drug had no effect when applied at T2 (the treatment that had the most robust effect on hemorrhage and cell death). On one hand, this observation suggests that the protective effect of bupivacaine at T2 is not tied to the change in spleen weight. On the other, the drop in spleen weight observed with repeated drug infusion suggests a heightened systemic response, which could counter the beneficial effect of prolonged drug infusion. Spleen weight is, however, an indirect measure of the immune response and further work is needed to assess how our experimental treatments affect the trafficking of cells to the site of injury.

### 4.3 Factors that may counter the beneficial effect of drug treatment at the site of injury

We had hypothesized that L4 bupivacaine would be effective based on our prior work examining the effect of another Nav blocker (lidocaine) on nociception-induced hemorrhage and work suggesting that a state of over-excitation may contribute to tissue damage after injury. Research has shown that dysfunction of Na + -K + ATPase after SCI depolarizes Nav channels, leading to further influx of Na + and ionic imbalance (40). Administration of a Nav blocker has been shown to spare white matter and improve functional recovery (41). Inward Nav-dependent current is also involved in microglial activation after SCI (42). These findings suggest that blocking Nav channels with bupivacaine could reduce excitotoxicity, and injury-related pathophysiology. However, our results did not align with this hypothesis. Results from hemoglobin, caspase-3, IL-1β, TNF-*α*, IgG, Iba-1, and GFAP found little effect of drug treatment at L4. It is possible, however, that the beneficial effect of local bupivacaine infusion was countered by its neurotoxic effect. Bupivacaine applied to the dorsal root ganglia for 3 h has been linked to mitochondrial calcium overload and increased production of reactive oxygen species (43), which could exacerbate neural damage at the site of injury (44). The implication is that dampening activity at the site of injury may be affecting *both* degenerative and reparative processes.

Given the importance of blood flow into the site of injury, local hemodynamics should also be considered. Clinical work has shown that SCI augments intrathecal pressure (ITP)—the pressure of the cerebrospinal fluid (CSF) within the subarachnoid space around the spinal cord, This increase correlates with poor recovery, and reducing ITP via a lumbar drainage benefits neurological recovery (45). While not measured in the current study, repeated drug infusions at L4 may have raised intrathecal pressure, attenuating the beneficial effect of bupivacaine treatment (relative to T2 administration). Assessing cerebrospinal fluid dynamics or ITP could help assure the safety of lumbosacral drug infusion.

### 4.4 Comparison to the effect of hypothermia

The present results are consistent with other work showing that reducing cellular activity with hypothermia can have a protective effect. It has been suggested that hypothermia acts by reducing metabolism, inflammation, and the engagement of apoptotic processes (46). Both systemic and local hypothermia have shown promising results in pre-clinical SCI models, yielding improvements in motor function and reduced tissue damage and inflammation (47). Similar findings have been reported for targeted temperature treatment in traumatic brain injury (TBI) (48).

A systematic review of human clinical work found that 58% of patients who presented with ASIA Grade A improved at least one grade after receiving local hypothermia (49). However, effectiveness may vary depending upon time of onset, duration, and injury severity. In addition, hypothermia can elicit shivering, discomfort, and stress, especially when applied systemically (50). While limited shivering can be treated with non-pharmacological interventions or acetaminophen, many patients must be moderately sedated with dexmedetomidine, a selective *α*-2 adrenergic receptor agonist with analgesic properties (51). In more severe cases of shivering, a general anesthetic (e.g., propofol) may be administered (50, 51). As discussed above, we have shown that inducing a sedated state with pentobarbital reduces hemorrhage and the loss of motor function after SCI (13). This raises the possibility that some of the benefits of hypothermia are attributable to the co-administration of a sedative. Indeed, in a murine model of spinal ischemic-reperfusion, pre-treatment with dexmedetomidine preserved hind-limb function and attenuated neuronal degeneration (52). In animal models of SCI, a post-operative systemic dose of dexmedetomidine preserved neuronal cell counts in the gray matter of the spinal cord when assessed 24 h after injury (53) and reduced pro-inflammatory profiles (54). These findings raise the possibility that the necessity of a sedative within clinical hypothermia could account for some of the benefit seen.

### 4.5 Neuroprotective effects of anesthesia in other model systems

As recently reviewed by Davis and Grau (55), anesthetics have been shown to have a neuroprotective effect in several injury models, including SCI, TBI, and stroke. Intrathecal administration of bupivacaine reduces microglial activation and neuropathic pain in a rat model of chronic constriction injury (56). In a spinal nerve ligation model, bupivacaine attenuates mechanical and cold allodynia (57). In a photochemical SCI model, intrathecal bupivacaine was shown to improve hindlimb dysfunction and restore somatosensory evoked potentials (58). Results from the current study are consistent with these findings, demonstrating the importance of targeting pain signaling or sympathetic activity in the acute periods of injury to minimize tissue loss and foster functional recovery.

### 4.6 Limitations and clinical application

A limitation of the current study concerns the timing of treatment initiation. In a clinical setting, delay in treatment initiation is inevitable – waiting for EMS to arrive on site and travel time to the hospital. In the present experiments, bupivacaine administration began immediately after injury, thus the therapeutic window is unknown. Further work is needed to address this issue and derive the optimal duration of treatment. In addition, research is needed to establish the long-term effects of bupivacaine treatment on tissue loss, locomotor function, and neuropathic pain. Finally, future studies should evaluate whether drugs with lower cardiotoxicity (e.g., the S-enantiomers levobupivacaine and ropivacaine) have a protective effect after SCI.

The current study was performed using male animals because the incidence of SCI in men is much higher. Further work is needed to determine whether rostral bupivacaine has a protective effect in female animals. For nociception-induced hemorrhage, similar results were obtained in female rats, though the magnitude of the effect observed varied with estrous cycle stage (59).

A surprising outcome concerns the beneficial effect of rostral bupivacaine: a finding that may implicate surviving fibers and brain-dependent processes. Further work is needed to determine the underlying mechanisms and whether treatments that inhibit brain function (e.g., general anesthesia, sedatives) have a comparable protective effect. This is particularly important given epidural application of an anesthetic rostral to injury may be difficult to perform in many scenarios and brings some risk.

## 5 Summary

The current study explored whether regional anesthesia has a protective effect after SCI. We found that application of bupivacaine rostral to injury for 3 or more hours reduces the acute effect of injury on hemorrhage, cell death, and locomotor function. Further work is needed to detail the underlying mechanisms and optimal treatment protocol.

## Data Availability

The raw data supporting the conclusions of this article will be made available by the authors, without undue reservation.
